# Auxin Distribution in Lateral Root Primordium Development Affects the Size and Lateral Root Diameter of Rice

**DOI:** 10.3389/fpls.2022.834378

**Published:** 2022-04-13

**Authors:** Tsubasa Kawai, Ryosuke Akahoshi, Israt J. Shelley, Takaaki Kojima, Moeko Sato, Hiroyuki Tsuji, Yoshiaki Inukai

**Affiliations:** ^1^Graduate School of Bioagricultural Sciences, Nagoya University, Nagoya, Japan; ^2^School of Agriculture and Environment, The UWA Institute of Agriculture, The University of Western Australia, Perth, WA, Australia; ^3^International Center for Research and Education in Agriculture, Nagoya University, Nagoya, Japan; ^4^Department of Crop Botany, Bangladesh Agricultural University, Mymensingh, Bangladesh; ^5^Kihara Institute for Biological Research, Yokohama City University, Yokohama, Japan

**Keywords:** lateral root, root development, dynamin-related protein, auxin distribution, rice

## Abstract

Lateral roots (LRs) occupy a large part of the root system and play a central role in plant water and nutrient uptake. Monocot plants, such as rice, produce two types of LRs: the S-type (short and thin) and the L-type (long, thick, and capable of further branching). Because of the ability to produce higher-order branches, the L-type LR formation contributes to efficient root system expansion. Auxin plays a major role in regulating the root system development, but its involvement in developing different types of LRs is largely unknown. Here, we show that auxin distribution is involved in regulating LR diameter. *Dynamin-related protein* (*DRP*) genes were isolated as causative genes of the mutants with increased L-type LR number and diameter than wild-type (WT). In the *drp* mutants, reduced endocytic activity was detected in rice protoplast and LRs with a decreased OsPIN1b-GFP endocytosis in the protoplast. Analysis of auxin distribution using auxin-responsive promoter *DR5* revealed the upregulated auxin signaling in L-type LR primordia (LRP) of the WT and the mutants. The application of polar auxin transport inhibitors enhanced the effect of exogenous auxin to increase LR diameter with upregulated auxin signaling in the basal part of LRP. Inducible repression of auxin signaling in the m*OsIAA3-GR* system suppressed the increase in LR diameter after root tip excision, suggesting a positive role of auxin signaling in LR diameter increase. A positive regulator of LR diameter, *OsWOX10*, was auxin-inducible and upregulated in the *drp* mutants more than the WT, and revealed as a potential target of ARF transcriptional activator. Therefore, auxin signaling upregulation in LRP, especially at the basal part, induces *OsWOX10* expression, increasing LR diameter.

## Introduction

The root system in rice comprises main roots, including an embryonic seminal root (SR) and shoot-borne crown roots (CRs), and their lateral roots (LRs). LRs are classified as S- and L-types based on their distinct morphological and anatomical characteristics (Kawata and Shibayama, 1965; Kono et al., 1972; Yamauchi et al., 1987). S-type LRs are thinner and shorter than L-types, and never produce higher order LRs. S-type LRs largely contribute to the hydraulic conductivity of the whole root system (representing water uptake ability) (Watanabe et al., 2020), while L-type LRs expand the root network in soils by producing higher-order LRs including both S- and L-types. The plastic development of LRs plays an important role for the adaptation to soil water fluctuation of rice (Suralta et al., 2018; Lucob-Agustin et al., 2021).

Plant hormone auxin plays a central role in LR formation. Under low auxin levels, Aux/IAA proteins repress auxin-responsive transcription (Ramos et al., 2001). Auxin perception by its receptor TIR1/AFB (Tan et al., 2007) promotes the ubiquitination of Aux/IAA by the TIR1/AFBs complex (Gray et al., 2001). The degradation of the ubiquitinated Aux/IAA by 26S proteasome allows auxin-responsive transcriptional regulation by ARF proteins (Gray et al., 2001). The core sequence GWPPV in domain II of Aux/IAA is required for the auxin-responsive ubiquitination of Aux/IAA, and mutations in the core sequence inhibit the auxin signaling transmission (Ramos et al., 2001). The gain-of-function mutants of Aux/IAA, from a stabilizing mutation in their domain II, decreased the number of LRs in *Arabidopsis* (e.g., Fukaki et al., 2002) and rice (e.g., Kitomi et al., 2012).

Auxin distribution regulated by its polar transport is crucial for root system development. The differential distribution of auxin is produced mainly by the AUXIN1/LIKE-AUX1 (AUX1/LAX) influx carrier and PIN-FORMED (PIN) efflux carriers in rice roots (Yu et al., 2016). The mutation in *OsAUX1* causes defects in LR formation and root hair elongation (Zhao et al., 2015). Additionally, the over-expression of *OsPIN1b* increases LR density (Xu et al., 2005) and the *Ospin1a Ospin1b* double mutant decreases lateral and crown root numbers (Li et al., 2019). In *Ospin2* mutant, the basipetal shifting of LR formation pattern was observed with higher auxin content in the SR tip (Inahashi et al., 2018). In *Arabidopsis*, auxin distribution changes dynamically during LR formation—auxin accumulates in pericycle cells that differentiate into LR primordia (LRP); then, gradual auxin distribution is established and maximized at the root tip by PIN proteins (Benková et al., 2003). A synthetic compound, *N*-1-naphthylphthalamic acid (NPA), has been used to study the role of polar auxin transport in plant tissue development, which directly binds to PIN auxin transporters and inhibits its IAA transport activity (Abas et al., 2021). In rice, NPA treatment inhibited LR formation with restricted lateral flow of auxin from the main roots to the LRs (Sreevidya et al., 2010). Therefore, proper auxin distribution by PIN proteins is important for LR formation.

Regulatory mechanisms of cellular localization of the PIN protein, crucial for directing auxin flow, have been well studied in *Arabidopsis* (Luschnig and Vert, 2014). During LR formation in *Arabidopsis*, PIN1 cellular localization changes from anticlinal to periclinal sides, which is crucial for establishing the auxin gradient and, thus, proper LR formation (Benková et al., 2003). Polar localization of PIN proteins is established by targeted exocytosis and endocytosis pathways. Guanine nucleotide exchange factors for ADP ribosylation factors (ARF-GEFs) mediate PIN exocytosis to the rootward plasma membrane in *Arabidopsis* roots, which is sensitive to a fungal toxin brefeldin A (BFA) (Steinmann et al., 1999; Geldner et al., 2003). BFA treatment inhibited the re-direction of PIN1 protein during LR formation in *Arabidopsis* (Benková et al., 2003). In rice, the mutation in ARF-GEF *CRL4/OsGNOM1* caused defects in crown and lateral root formation (Kitomi et al., 2008; Liu et al., 2009). Clathrin-mediated endocytosis (CME) has a central role in PIN protein endocytosis (Dhonukshe et al., 2007; Kleine-Vehn et al., 2011; Kitakura et al., 2011). Dynamin is a large GTPase and functions in membrane scission of the clathrin-coated vesicles. In plants, dynamin-related proteins DRP1 and DRP2 are involved in CME (Fujimoto et al., 2010). In *Arabidopsis*, loss-of-function of *DRP1A* caused defects in auxin-related developmental phenotypes (Mravec et al., 2011). Regulatory mechanisms of auxin distribution and its function for root formation have been well-studied; however, the involvement of auxin distribution in the LR type determination in rice is poorly understood.

Lateral roots are derived from the pericycle and endodermal cells of the main roots in rice. The LR type is characterized during the LRP stage with distinct size of meristem formed in the two types (Kawata and Shibayama, 1965; Kawai et al., 2022a). Root tip excision was used to analyze the molecular mechanisms underlying the control of LRP size, which promotes L-type LR formation (Sasaki et al., 1984; Kawai et al., 2017, 2022b). Transcriptome analysis suggested the upregulation of auxin signaling in L-type LRP than S-types, which possibly induces the expression of *OsWOX10*, a positive regulator of LR diameter (Kawai et al., 2022a). In another study, a rice mutant, *weg1*, was selected for its promoted L-type LR formation with a wavy main root phenotype (Lucob-Agustin et al., 2020). Observation of auxin signaling using a fluorescent protein driven by the auxin-responsive promoter *DR5* revealed the upregulation of auxin signaling on the convex side of curvature where L-type LRs develop (Lucob-Agustin et al., 2020). The secondary LR development is positively regulated by auxin and inhibited by strigolactone signaling (Sun et al., 2019). These studies suggest the involvement of auxin in determining the LR type. However, how the two types of LRP differ in spatiotemporal auxin distribution remains unclear.

In this study, we isolated novel rice mutants with thicker LRs than wild-type (WT). Map-based cloning revealed *DRP* family genes as the causative genes of mutants. The auxin signaling distribution in LRP revealed different distribution patterns in the WT and the mutants. Exogenous treatment of auxin and its polar transport inhibitors further suggested the regulation of LR diameter through auxin distribution in LRP.

## Materials and Methods

### Plant Material and Growth Conditions

*N*-methyl-*N*-nitrosourea (MNU) mutagenized population of rice plants (*Oryza sativa* L. cv. “Taichung 65”) was obtained using the panicle-dipping method (Sato and Omura, 1979). Treated panicles were harvested to obtain M_1_ seeds, which were grown to obtain M_2_ seeds. Rice seedlings were grown hydroponically. Rice seeds were pregerminated in water mixed with fungicide [0.25% (w/v) benomyl benlate; Sumitomo Chemical Co., Japan] and placed in a growth chamber (MLR-351; Sanyo, Japan) at 28°C with continuous light for 3 days. Germinated seeds were rinsed with tap water and transferred onto plastic nets floating on 10% nutrient solution (Colmer, 2003) aerated with a pump (OX-30; Tetra-Japan, Japan) in a 9-L black plastic box (32 cm height × 19 cm length × 19 cm width). For map-based cloning, seeds of the WT, mutants, and F_2_ plants derived from crosses between the mutants and *O. sativa* “Kasalath” were grown for 2 weeks. For inducing L-type LR formation in *DR5-NLS-3xVENUS* and *pOsAct1*-m*OsIAA3-GR*, CRs of approximately 10 cm length were excised at 5 mm from the root tip with a cutting knife. To analyze the LR formation in *pOsAct1*-m*OsIAA3-GR*, transgenic plants were transferred prior to the root tip excision to the solution containing 50 μM dexamethasone (DEX) for different durations. The transgenic plants after the root tip excision were grown under the DEX condition for another 3 days. For chemical treatments, 4-day-old WT and mutants’ seedlings were transferred to tap water filtered by a water purifier (MX600; TORAY Industries, Japan) containing IAA and/or inhibitor (1 μM NPA or BFA) and grown for another 10 days.

### Morphological Characterization

To characterize LR phenotypes in the WT and mutants, the SRs of 10-day-old seedlings were sampled and fixed in FAA solution [5 formaldehyde, 5 acetic acid, 63% ethanol (v/v)]. The LR number on sampled SRs in three diameter classes (<100 μm, S-type; ≥ 150 μm, L-type; 100–150 μm, intermediate type) was counted. The density of LRs was calculated as the LR number divided by the length of SR formed LRs. The diameter and length were measured in LRs emerged on the basal part of SRs (2–4 cm). The root segments were fully extended on a slide and images were taken with a digital camera (D90; Nikon, Japan). Diameters of the first-order LRs were measured in the basal region using a microscope (IX71; Olympus, Japan) and a micrometer. Lengths of the first-order LRs were measured from images using LIA for Win32.^1^

To characterize LR phenotypes in the WT and mutants with chemical treatments, SR length and the length of LR-formed region were recorded when the seedlings were transferred to the treatment solution. SR segments corresponding to the region without LRs at transfer were sampled for LR measurement.

### Map-Based Cloning, Plasmid Constructs, and Plant Transformation

The linkage analysis of SSR (simple-sequence repeats) markers was performed using the F_2_ plants derived from crosses between each mutant and “Kasalath.” For T12-3, T3-2, and T12-36, 779, 13, and 443 F_2_ plants that exhibited the mutant phenotype were used for the linkage analysis, respectively. To complement the mutation, *pOsDRP1C-OsDRP1C-GFP* construct was transformed into T12-3. The genomic sequence of *OsDRP1C* from approximately –3 kb to –1 bp (*OsDRP1C* translation start site: + 1 bp) and the coding sequence of *OsDRP1C* were amplified from “Taichung 65” genomic and complementary DNA, respectively, and cloned into pENTR/D-TOPO^®^. The *pOsDRP1C-OsDRP1C* fragment was then transferred into the pGWB4 vector (Nakagawa et al., 2007) using the Gateway LR reaction. For the transient expression of *OsPIN1b-GFP* in rice protoplast, *p35S-OsPIN1b-GFP* was transformed into protoplasts of the WT and mutants. The coding sequence of *OsPIN1b* was amplified from “Taichung 65” complementary DNA, fused to *GFP* derived from the pGWB4 vector, then cloned into pGWB502Ω vector harboring *2* × *35S* promoter with translation enhancer (Nakagawa et al., 2007). The *35S-OsPIN1b-GFP* fragment with NOS terminator was then transferred into pENTR/D-TOPO^®^. The *DR5-NLS-3xVENUS* was constructed as previously described (Lucob-Agustin et al., 2020). The *pOsAct1*-m*OsIAA3-GR* was constructed as previously described (Nakamura et al., 2006). Briefly, the coding sequence of *OsIAA3* (Os12g0601400) was amplified and a nucleotide substitution in domain II (P58L) by PCR was introduced and then fused to the *pAct-GR*-Hm_2_ vector harboring rice *Actin 1* promoter and the steroid-binding domain of the human GR. The primers used in this study are listed in Supplementary Table 1. The fusion constructs were introduced into *Agrobacterium tumefaciens* strain EHA105 by electroporation. The *Agrobacterium*-mediated transformation of rice was then performed as described elsewhere (Hiei et al., 1994; Ozawa, 2009). Transgenic plants were selected on Murashige and Skoog (MS) medium containing 50 mg/l hygromycin at 30°C.

### Microscopic Observation

Crown root sections were sampled to observe *DR5-NLS-3xVenus* expression in the LRP of the WT and mutants. The samples were fixed in 4% paraformaldehyde (w/v) and stained with 5 μg/ml DAPI (4′,6-Diamidino-2-phenylindole Dihydrochloride; Wako, Japan) for 1 h, then cleared with iTOMEI (TCI, Japan) (Sato et al., 2022; Sakamoto et al., 2022), or embedded in a 3% (w/v) agar medium then cross-sectioned into 50 μm thick slices with a linear slicer (DYK-1000N; Dosaka EM Co., Japan). The samples were viewed under a confocal laser scanning microscope (FV3000; Olympus, Japan). Venus fluorescence was excited at 488 nm and detected at 510–530 nm, while DAPI or autofluorescence of the root samples was excited at 405 nm and detected at 450–470 nm. We have confirmed that the WT and mutants’ phenotypes were not affected by the transformation.

### Expression Analysis

Total RNA was extracted from SR segments including the earlier stage of LRP (Part 3 in Supplementary Figure 4A) in 5-day-old seedlings using the NucleoSpin^®^ RNA Plant Kit (Macherey-Nagel, Germany) following the manufacturer’s instructions. Quantitative reverse-transcription PCR (qRT-PCR) was performed using the One-Step SYBR PrimeScript RT-PCR Kit II (Perfect Real-Time) (TaKaRa Bio, Japan) and StepOnePlus Real-Time PCR (Life Technologies, United States). The expression was normalized to that of *OsUBQ5* (Os03g0234200). All primers are listed in Supplementary Table 1. The expression of *OsDRP* family genes was compared using the RNA-seq data in S- and L-type LRP with four biological replications (Kawai et al., 2022a). The read counts were normalized by coding sequence length (per 1 kb) for all genes in each replicate to calculate transcript per million (TPM). The normalized read counts were further normalized by summing the normalized counts of all genes (per million reads). Means of the calculated TPMs of four biological replications were used to compare the expression levels between S- and L-type LRP. The significance of the difference between S- and L-type LRP was determined by *Padj* values calculated by DESeq2 (Love et al., 2014).

### Transient Expression in Rice Protoplast

For the protoplast isolation, rice seedlings of the WT and mutants were grown in 1/2 MS medium. For the first 3 days, rice seeds were placed on the medium in the growth chamber at 28°C with continuous light to promote seed germination, then transferred to the dark condition to reduce the autofluorescence from chloroplast. Protoplasts were isolated from the stem and sheath of 2-week-old seedlings as previously described (Zhang et al., 2011). Briefly, 20 μg of the plasmid DNA was mixed with 100 μl protoplasts (2 × 10^5^ cells), then transformed *via* PEG. The transformed protoplasts with *OsPIN1b-GFP* were treated with 10 μM FM4-64 (Setareh Biotech, United States) for 30 min at room temperature, then viewed under the confocal laser scanning microscope. GFP fluorescence was excited at 488 nm and detected at 500–540 nm, while FM4-64 was excited at 561 nm and detected at 570–620 nm.

### FM4-64 Uptake Assay in Rice Roots

To test the endocytic activity, WT and mutants’ seedlings were grown in the 10% nutrient solution for 6 days, then transferred to the solution containing 10 μM FM4-64. After 30 min treatment of FM4-64 at room temperature, S-type LRs were sampled and immediately viewed under the confocal laser scanning microscope as described above.

### Electrophoresis Mobility Shift Assay

The binding of OsARF19, preferentially expressed in LRP (Yamauchi et al., 2019), to auxin response elements (AuxREs) on *OsWOX10* promoter was analyzed by performing an electrophoresis mobility shift assay (EMSA). The sequence encoding the N-terminal 265 amino acids of OsARF19, including the B3 DNA-binding domain (DB), that was optimized for the codon usage of *E. coli*, was fused to pET32a. The OsARF19-DB was expressed in *E. coli* BL21(DE3), followed by purification with TALON metal affinity resin (Clontech, United States) following the manufacturer’s instructions. To prepare DNA probes, the 59 or 60-bp oligonucleotides were labeled with Cy5 fluorescence dye using Klenow fragment (TaKaRa Bio, Japan) and purified on a column (NucleoSpin^®^ Gel and PCR Clean-up; Macherey-Nagel, Germany) following the manufacturer’s instructions. The DNA-binding reaction was performed at 4°C for 30 min in PBS (–) (137 mM NaCl, 8.10 mM Na_2_HPO_4_, 2.68 mM KCl, 1.47 mM KH_2_PO_4_; pH 7.4) + 1 mM 2-Mercaptoethanol with 25.3 nM probe with 0, 0.5, or 1 μM recombinant OsARF19-DB, and subjected to EMSA with 5% polyacrylamide gels in 0.5 × TBE buffer at 4°C. The Cy5-labeled probes were analyzed by Typhoon FLA9000 (GE Healthcare, United States).

### Statistical Analysis

Differences in morphological and anatomical characteristics, and gene expression between groups were compared using two-tailed Student’s *t*-test or one-way ANOVA and a multiple-comparison Tukey’s test using the glht function from multcomp package in R (R Core Team, 2020).

## Results and Discussion

### Identification of Mutants With Promoted L-Type Lateral Root Formation

**FIGURE 1:**
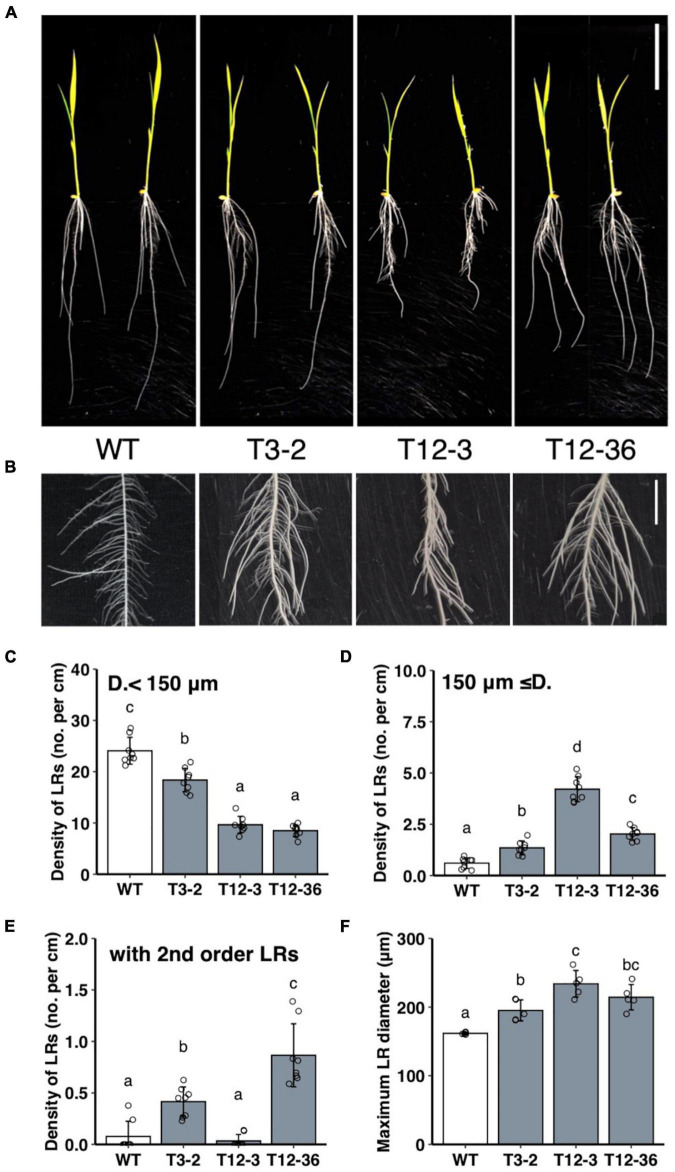
Analysis of lateral root (LR) formation on seminal roots (SRs) in 10-day-old seedlings of the wild-type (WT) rice and mutants (T3-2, T12-3, and T12-36). **(A)** Shoot and root phenotypes. Two representative plantlets are shown. Scale bar = 5 cm. **(B)** LR phenotypes on SRs. Scale bar = 1 cm. **(C,D)** Density of LRs in two different diameter classes. D., LR diameter. **(E)** Density of first-order LRs forming second-order LRs. **(F)** Maximum LR diameter. Values represent mean ± SD [*n* = 8 **(C–E)** and 5 **(F)** independent biological replicates]. Different letters indicate significant differences among genotypes (*P* < 0.05).

MNU-mutagenized populations were analyzed for their root formation to study the molecular mechanisms underlying the control of LRP size. The three mutants had fewer thinner LRs (< 150 μm in diameter) and thicker LRs (≥150 μm in diameter) than the WT (Figures 1A–D). Total LR number decreased in the three mutants (Supplementary Figure 1A). The number of LRs forming second-order LRs increased in T3-2 and T12-36 compared to the WT (Figure 1E). Furthermore, the LR diameter of the thickest LRs increased in the three mutants (Figure 1F). The lengths of the thinner LRs decreased in T3-2 and T12-3, and the length of the thicker LRs also decreased in T12-3, compared to the WT (Supplementary Figures 1B,C). The mutants also had a shorter SR than the WT, with the shortest SR in T12-3 (Supplementary Figure 1D). In T12-3, the CR number slightly increased than the WT (Supplementary Figure 1E). The mutants had shorter leaf lengths than the WT (Supplementary Figure 1F).

### Isolation of the Mutants’ Causative Genes

**FIGURE 2:**
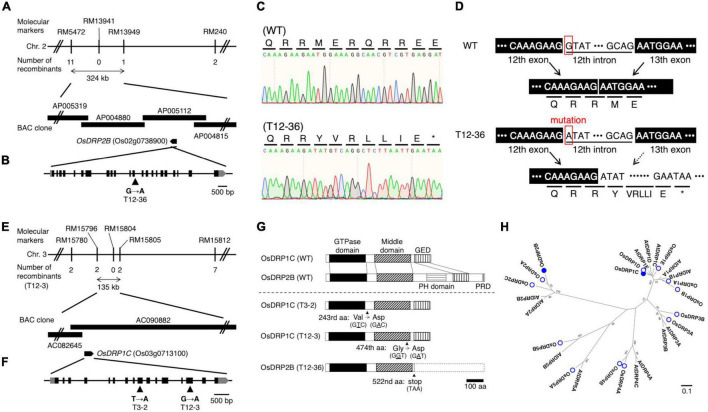
Map-based cloning of the causative genes of the mutants. **(A,E)** Linkage and physical map of the causative gene locus in **(A)** T12-36 and **(E)** T12-3. The vertical bars represent molecular markers, and the numbers of recombinant plants are indicated below the linkage map. **(B,F)** Structure of **(B)** *OsDRP2B* (Os02g0738900) and **(F)** *OsDRP1C* (Os03g0713100). Black and gray boxes indicate exons and untranslated regions (UTRs), respectively. Single nucleotide mutation at position **(B)** 4,537 bp of *OsDRP2B* and **(F)** 2,708 bp (T3-2) and 4,314 bp (T12-3) of *OsDRP1C* in the open reading frame (ORF) (indicated by arrowheads). **(C)** Sanger sequencing analysis around the splicing junction of 12th intron in *OsDRP2B* cDNA in the wild-type (WT) and T12-36. **(D)** *OsDRP2B* mRNA structures and amino acid sequences in WT and T12-36 mutant. **(G)** Domain structure of OsDRP1C and OsDRP2B in WT and the mutants. GED, GTPase-effector domain. PH, Pleckstrin homology. PRD, Proline-rich domain. **(H)** Phylogenetic tree constructed using the full-length amino acid sequences of DRP family proteins in rice and *Arabidopsis* using MEGA7. Numbers on branches are the 1,000-bootstrap values. Rice DRP proteins, open circles; proteins encoded by the causative genes of mutants, filled circles. Accession numbers of proteins are in Supplementary Table 3.

Linkage analyses of SSR markers were undertaken using the F_2_ plants derived from crosses between each mutant and “Kasalath” to identify the causative genes of mutants. The root phenotype of the mapping populations was segregated in a 3:1 WT:mutant ratio in the linkage analyses. In T12-36, the linkage analysis revealed the locus on chromosome 2 between RM5472 and RM13949 (∼324 kb) (Figure 2A). This region contains around 50 genes including *OsDRP2B* (*Dynamin-related protein 2B*, Os02g0738900). Comparison of the sequences between the WT and T12-36 revealed a single nucleotide substitution from G to A in the 12th intron (Figure 2B). As the mutated site was likely important for splicing in *OsDRP2B*, we compared the mRNA sequence by synthesizing cDNA from the WT and mutant. The splicing of the 12th intron was defective in T12-36, causing a stop codon after the 12th exon (Figures 2C,D). DRP2 proteins have five functional domains like the animal dynamin, while DRP1 proteins lack a PH (Pleckstrin homology) domain and a PRD (Proline-rich domain), which function in association with the cell membrane and actin (Schmid and Frolov, 2011; Figure 2G). T12-36 might produce a truncated OsDRP2B protein without a PH domain, GED (GTPase-effector domain), and PRD (Figure 2G). In previous studies, *OsDRP2B* was isolated as the causative gene of *brittle culm 3* (*bc3*) mutant (Hirano et al., 2010; Xiong et al., 2010). The *bc3* mutant exhibited brittle culm and shorter shoot and root phenotypes (Hirano et al., 2010; Xiong et al., 2010), as observed in T12-36 in the present study (Figure 1 and Supplementary Figure 1), indicating that T12-36 is an allelic mutant of the previously reported *bc3* mutant.

In T12-3, the linkage analysis revealed the locus on chromosome 3 between RM15796 and RM15805 (∼135 kb) (Figure 2E). This region contains 14 genes including *OsDRP1C* (*Dynamin-related protein 1C*, Os03g0713100). Comparing the WT and mutant sequences revealed a single nucleotide substitution, from G to A in the 14th exon (Figure 2F). In T3-2, a rough mapping revealed the locus between RM15414 and RM3199 (∼8 Mb), which contains the mapped region in T12-3. Therefore, we compared the *OsDRP1C* sequence between the WT and T3-2, detecting a single nucleotide substitution from T to A in the 9th exon (Figure 2F). In T12-3, the nucleotide substitution causes Gly to Asp amino acid substitution in the middle domain, mediating the protein interaction between dynamins (Ramachandran et al., 2007), while the mutation in T3-2 causes Val to Asp amino acid substitution between GTPase and middle domain of OsDRP1C (Figure 2G). The more distinct phenotypes in T12-3 than T3-2 (Figure 1 and Supplementary Figure 1) are possibly associated with the mutation sites in these two mutants. The introduction of ∼3 kb promoter and the coding sequence of *OsDRP1C* into T12-3 complemented the mutant phenotype (Supplementary Figure 2), confirming that *OsDRP1C* is the causative gene of T12-3.

In rice, there are 14 dynamin family proteins, classified into six groups (Figure 2H). The DRP1 and DRP2 proteins function in post-Golgi trafficking in *Arabidopsis* (Fujimoto et al., 2010). OsDRP2B localized in the plasma membrane, clathrin-mediated vesicles, and *trans*-Golgi network, involving in the endocytic pathway (Xiong et al., 2010). Co-expression of *OsDRP1C* and *OsDRP2B* was detected in a rice co-expression network database (RiceFREND; Sato et al., 2013a; Supplementary Table 2). In a public rice gene expression database (RiceXPro; Sato et al., 2013b), *OsDRP1C* and *OsDRP2B* had similar expression patterns among plant organs and tissues with relatively higher expression in leaf sheath and stems, roots, and reproductive tissues (Supplementary Figure 3). The *OsDRP1C* and *OsDRP2B* genes along the SR axis had similar expressions, with relatively higher expressions in the LR emerged region (Supplementary Figures 4A–C). Based on the RNA-seq result in S- and L-type LRP in the WT (Kawai et al., 2022a), *OsDRP1C* and *OsDRP2B* were highly expressed in S- and L-type LRP, relative to other *OsDRP* genes (Supplementary Figure 4D). Thus, these two rice DRPs may cooperate in CME, involving in LR diameter control.

### Altered PIN Protein Localization With Reduced Endocytic Activity in *Dynamin-Related Protein* Mutants

**FIGURE 3:**
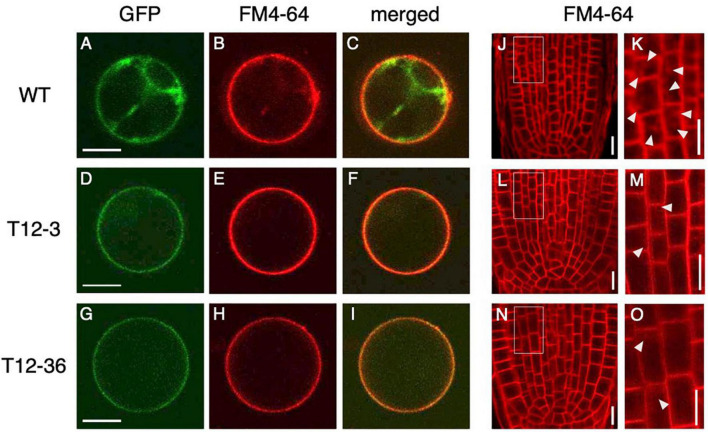
Analysis of endocytic activity and OsPIN1b localization in wild-type (WT) and the mutants. **(A–I)** Rice protoplasts expressing *35S-OsPIN1b-GFP* stained with an endocytic tracer FM4-64 in WT **(A–C)**, T12-3 **(D–F)**, and T12-36 **(G–I)**. GFP **(A,D,G)**, FM4-64 **(B,E,H)** signals, and the merged images **(C,F,I)** are shown. **(J–O)** FM4-64 uptake in root tips of S-type LRs in WT **(J,K)**, T12-3 **(L,M)**, and T12-36 **(N,O)**. Root tips **(J,L,N)** and enlarged views of the boxed area **(K,M,O)** are shown. Arrowheads point endocytic compartments in cortical cells. Scale bars = 10 μm.

Because dynamin functions in the CME of auxin transporter PIN proteins (Mravec et al., 2011), the mutations in *DRP*s might affect cellular localization of PIN proteins and hence auxin distribution in LRP. We first analyzed PIN localization and the endocytic activity in rice protoplast of the WT and *drp* mutants. OsPIN1b, which was highly expressed in both S- and L-type LRP among *PIN* family genes in the WT (Supplementary Figure 5), was fused to GFP and overexpressed in rice protoplast under the 35S promoter. Endocytic activity was monitored with a fluorescent endocytic tracer FM4-64. In WT, OsPIN1b-GFP was detected at plasma membrane and intracellular compartments (Figure 3A). FM4-64 was overlapped with the OsPIN1b-GFP signal (Figures 3B,C), confirming its localization in the endocytic pathway. In contrast, OsPIN1b-GFP signal was detected at the plasma membrane but absent from intracellular spaces in T12-3 and T12-36 with reduced FM4-64 uptake (Figures 3D–I). These observations suggest that OsDRP1C and OsDRP2B function in the endocytosis of OsPIN1b proteins in rice protoplast.

To further analyze if endocytic activity was affected in the roots of *drp* mutants, FM4-64 uptake was analyzed in the roots of WT and *drp* mutants. In WT, FM4-64 was detected at plasma membrane and endosomal compartments in the root tips of S-type LRs (Figures 3J,K). In T12-3 and T12-36, FM4-64 was detected at plasma membrane, but its intracellular uptake was reduced (Figures 3L–O). Therefore, the mutations in *OsDRP1C* and *OsDRP2B* reduces endocytic activity, affecting PIN protein localization in rice roots.

### Altered Auxin Distribution in Lateral Root Primordia of *Dynamin-Related Protein* Mutants

**FIGURE 4:**
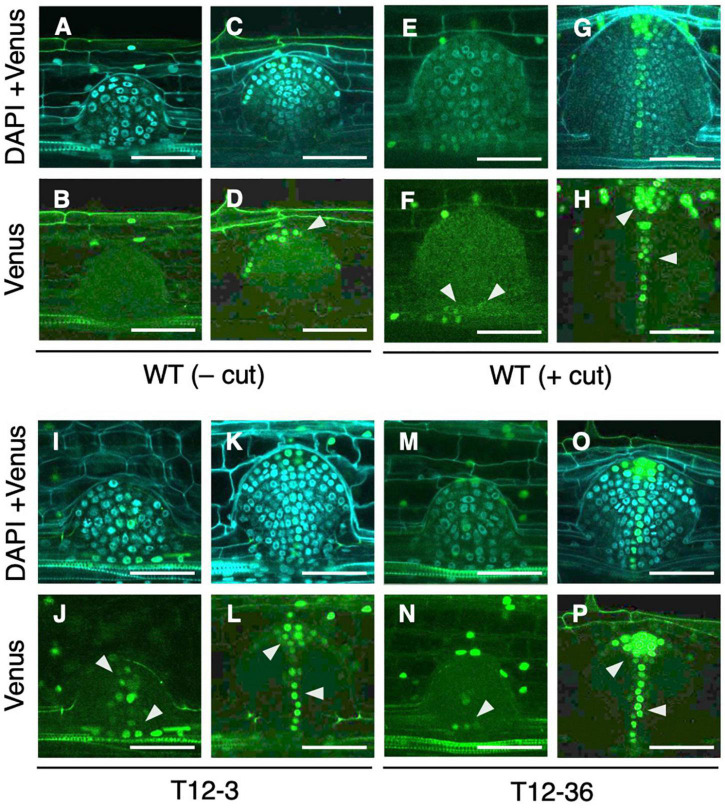
Analysis of auxin signaling distribution in lateral root primordia (LRP) of wild-type (WT) and the mutants. LRP observed in transverse section of crown roots expressing *DR5-NLS-3xVENUS* in the background of WT **(A–H)**, T12-3 **(I–L)**, and T12-36 **(M–P)**. In the WT, S-type LRP without root tip excision (– cut; **A–D**) and L-type LRP with root tip excision (+ cut); 15 h in **(E,F)**; 24 h in **(G,H)** are shown. In the mutants, L-type LRP without root tip excision are shown. For each genotype/treatment, LRP before **(A,B,E,F,I,J,M,N)** and after **(C,D,G,H,K,L,O,P)** meristem formation is shown. Venus signals and its merged images with DAPI signals are shown. Arrowheads point the Venus signals in LRP. Scale bars = 50 μm.

Next, we analyzed whether auxin signaling changes in the LRP of the *drp* mutants using a *DR5-NLS-3xVENUS* construct transformed into T12-3 and T12-36. In the S-type LRP of the WT, Venus signal was detected after meristem formation in root cap cells (Figures 4C,D), but not before meristem formation (Figures 4A,B). Because the WT produced fewer L-type LRs in the control (Figures 1B,D), root tip excision was conducted on CRs to analyze auxin signaling in L-type LRP. In the L-type LRP of the WT, Venus signal was detected in the basal part before the meristem formation, and in the root tip and stele cells in the established meristem (Figures 4E–H). In the mutants, Venus signal was also detected from an early stage in the basal part of LRP (Figures 4I,J,M,N), and in the root tip and stele cells after meristem formation (Figures 4K,L,O,P). Therefore, auxin signaling is upregulated in an early stage of L-type LRP in the WT after root tip excision and in *drp* mutants.

In *Arabidopsis*, PIN1 localization shifts from anticlinal sides toward the tip of LRP during LR formation, forming auxin maxima in the tip (Tanaka et al., 2013). In contrast, the mutations in *BEN1* and *BEN2*, involved in intracellular trafficking of PINs, caused less PIN relocation toward the LRP tip, resulting in broader *DR5* expression and wider LRP than the WT (Tanaka et al., 2013). Therefore, altered auxin distribution could affect LRP diameter. It is noteworthy that the expression of *DRP* family genes did not differ between the S- and L-type LRP (Supplementary Figure 4D). Thus, factor(s) other than DRPs might regulate auxin distribution and LRP size in the WT. It was also suggested that auxin transport and *de novo* biosynthesis is involved in L-type LR formation after root tip excision (Kawai et al., 2022a).

### Inhibition of Polar Auxin Transport Enhanced L-Type Lateral Roots Formation

**FIGURE 5:**
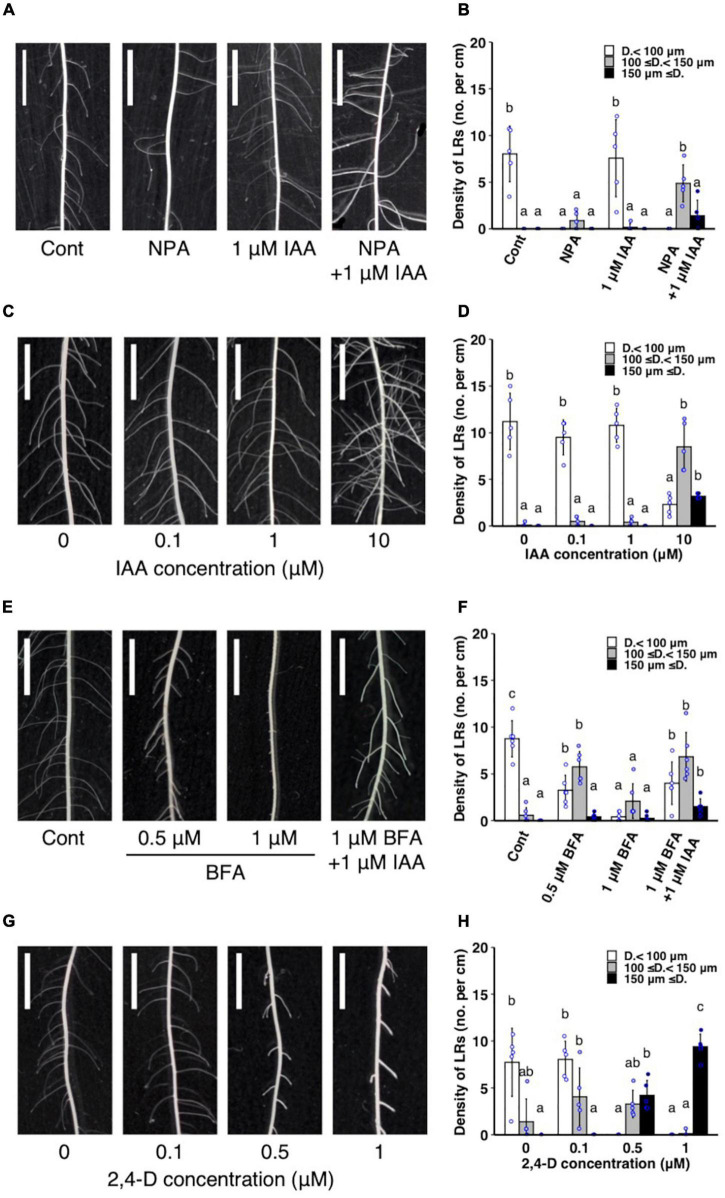
Analysis of the effect of exogenous auxin and chemical inhibitors on lateral root (LR) formation on seminal roots in wild-type rice. **(A,B)** Treatment of IAA and a polar auxin transport inhibitor NPA. **(C,D)** IAA treatment. **(E,F)** Treatment of IAA and a vesicle trafficking inhibitor BFA. **(G,H)** 2,4-D treatment. **(A,C,E,G)** LR formation in control and treated plants. Scale bars = 5 mm. **(B,D,F,H)** Density of LRs in different diameter classes. D., LR diameter. Values represent mean ± SD (*n* = 5 independent biological replicates). Different letters indicate significant differences among treatments (*P* < 0.05).

The WT plants were treated with auxin transport inhibitors with or without IAA co-treatment to further analyze whether the altered auxin transport is involved in LR diameter control. A single NPA treatment reduced the number of thinner LRs (<100 μm) (Figures 5A,B) and did not increase LR diameter (Supplementary Figure 6A). A single IAA treatment induced thicker LRs (≥150 μm) at a high concentration (10 μM) (Figures 5C,D, and Supplementary Figure 6B). In contrast, the co-treatment of a lower concentration of IAA (1 μM) and NPA increased LRs with intermediate diameter between S- and L-types (100–150 μm) (Figures 5A,B) and LR diameter (Supplementary Figure 6A). Similarly, a vesicle trafficking inhibitor BFA (1 μM) with 1 μM IAA increased the thicker LRs (≥ 150 μm) (Figures 5E,F). Furthermore, 0.5 μM BFA increased the LRs with intermediate diameter (100–150 μm) and LR diameter although 1 μM BFA inhibited LR formation (Figures 5E,F and Supplementary Figure 6C). Therefore, the inhibition of polar auxin transport enhanced the effect of exogenous auxin to induce L-type LRs. The 2,4-D is an artificial auxin which moves in plant tissues independent of the polar auxin transport (Sugawara et al., 2015). The 2,4-D treatment induced thicker LRs (≥ 150 μm) at a lower concentration (0.5 μM) than in IAA treatment (Figures 5G,H and Supplementary Figure 6D), further suggesting that non-directional movement of auxin could increase LR diameter more effectively than polar auxin transport. To analyze if the auxin sensitivity is altered in *drp* mutants, T12-3 and T12-36 were treated with IAA. The lower concentration of IAA (1 μM) significantly increased thicker LRs (≥ 150 μm) in the mutants but not in WT (Supplementary Figure 6E), suggesting the higher auxin sensitivity in the *drp* mutants than WT.

**FIGURE 6:**
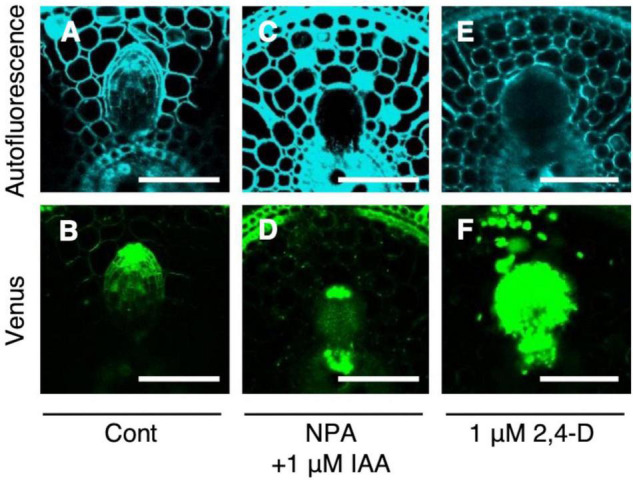
Analysis of auxin signaling distribution in lateral root primordia (LRP) of wild-type (WT) rice treated with auxin and chemical inhibitor. LRP observed in cross-section of crown roots expressing *DR5-NLS-3xVENUS* in the background of WT in the **(A,B)** control, **(C,D)** co-treatment of NPA and IAA, and **(E,F)** 2,4-D treatment. Autofluorescence and Venus signals are shown. Scale bars = 100 μm.

Auxin signaling was observed after the IAA and NPA treatment using *DR5-NLS-3xVENUS* in the WT. After the co-treatment of 1 μM IAA and NPA, Venus signal was detected in the apical and basal part of LRP (Figures 6C,D), but only in apical parts in the control (Figures 6A,B). After the 2,4-D treatment, Venus signal was detected in whole LRP (Figures 6E,F). It is noteworthy that auxin signaling was also upregulated at the basal part of L-type LRP in WT after root tip excision and in *drp* mutants (Figure 4), suggesting that auxin accumulation at the basal part of LRP increases LR diameter.

### Repressed L-Type Lateral Roots Formation After Root Tip Excision by Inducible Suppression of Auxin Signaling

**FIGURE 7:**
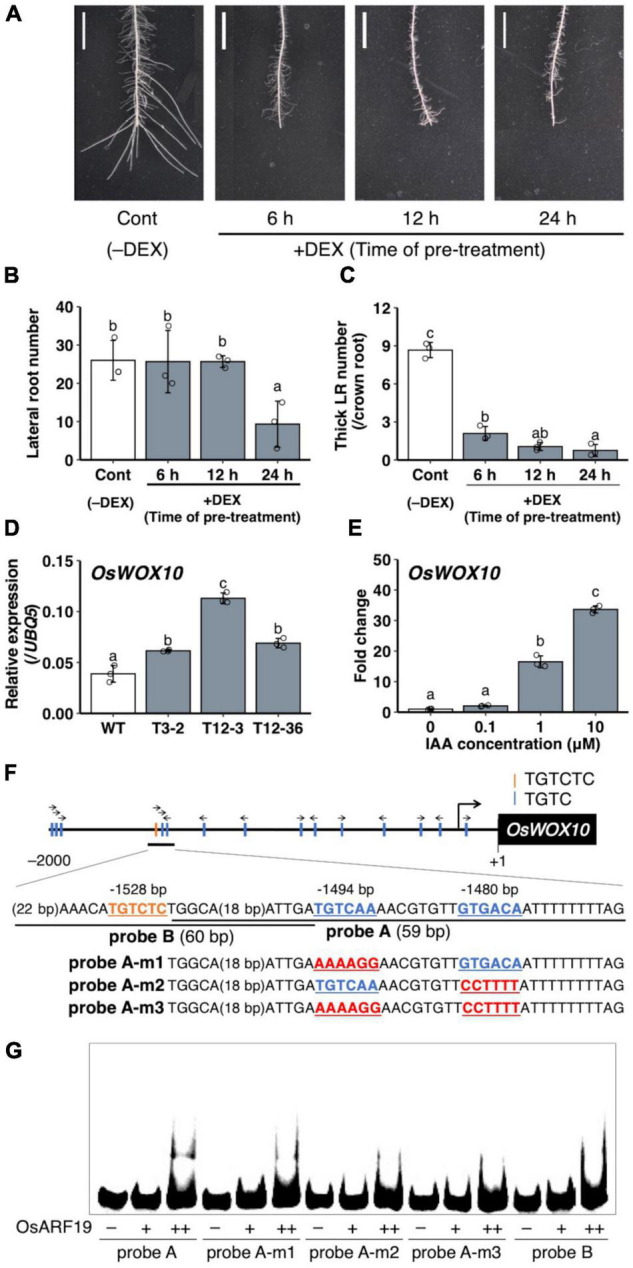
Analysis of the involvement of auxin signaling for lateral root (LR) diameter control and *OsWOX10* expression. **(A–C)** Root tip excision in *pOsAct1*-m*OsIAA3-GR* transgenic plants in the background of Nipponbare with or without the m*OsIAA3* activation by dexamethasone (DEX). DEX was pre-treated before root tip excision for different durations. **(A)** LR formation on crown roots (CRs) 3 days after root tip excision. Scale bars = 1 cm. **(B)** Total LR number in 0–1 cm from the cut site. **(C)** Number of thicker LRs (≥ 150 μm) per CR in **(A)** (*n* = 3 biological replicates). **(D)** *OsWOX10* expression levels in seminal roots with developing LRP in wild-type (WT) and the mutants. **(E)** *OsWOX10* expression levels after 1 h of IAA treatment with graded concentrations (*n* = 3 biological replicates). Values represent mean ± SD. Different letters indicate significant differences among groups (*P* < 0.05). **(F)** Schematic diagram showing auxin-response elements located in 2 kb upstream of *OsWOX10* and probe sequences used in **(G)**. **(G)** Electrophoresis mobility shift assay (EMSA) in recombinant OsARF19 DNA-binding domain with Cy5-labeled probes containing candidate sequences in *OsWOX10* promoter.

To analyze the role of the upregulated auxin signaling in L-type LRP for LR diameter increase, auxin signaling was suppressed by expressing mutagenized OsIAA3 (mOsIAA3), which is not degraded by auxin, using a steroid hormone-inducible system (Nakamura et al., 2006). In this system, the over-expressed m*OsIAA3* by rice *Actin 1* gene promoter is activated by DEX application. In the transgenic Nipponbare plants harboring *pOsAct1*-m*OsIAA3-GR*, 24 h pre-treatment of DEX decreased LR number after root tip excision (Figures 7A,B). The shorter pre-treatments of DEX (6 and 12 h) did not affect the LR number; however, it decreased the number of thicker LRs that emerged after root tip excision (Figures 7A–C), suggesting the positive role of auxin signaling in LR diameter increase after root tip excision.

### Regulation of a Positive Regulator of Lateral Roots Diameter *OsWOX10* Through Auxin Signaling

A *WUSCHEL-related homeobox* gene, *OsWOX10*, was identified as a positive regulator of LR diameter, upregulated in L-type LRP in WT after root tip excision (Kawai et al., 2022a). To examine whether *OsWOX10* is involved in thicker LR formation in *drp* mutants, *OsWOX10* expression was measured by qRT-PCR. The *drp* mutants had higher *OsWOX10* expression than the WT in SR segments that include developing LRP (Figure 7D). It is noteworthy that the *OsWOX10* expression level was positively correlated with the maximum LR diameter among the WT and mutants (Figure 1F), suggesting that increased *OsWOX10* expression increases LR diameter in *drp* mutants. *OsWOX10* expression increased after 1 h of the exogenous IAA treatment in WT roots in a dose-dependent manner (Figure 7E). Because 15 AuxREs (TGTCNN) exist in 2 kb upstream region of *OsWOX10* (Figure 7F), the binding of an ARF transcriptional factor, OsARF19, to the AuxREs was tested by performing an EMSA. OsARF19 was selected for its transcriptional activation activity and the positive role in LR formation in rice with the higher expression levels in developing LRP among rice *ARF* family genes (Yamauchi et al., 2019). The OsARF19 bound to probe A containing two AuxREs facing each other (TGTCAA/GTGACA) but not to probe B including an AuxRE (TGTCTC) (Figure 7G). Mutating one of the AuxREs in probe A weakened the binding (Figure 7G). Therefore, *OsWOX10* is a potential target of OsARF19.

Notably, auxin signaling was upregulated in LRP, including the basal part in WT after root tip excision and in *drp* mutants (Figure 4), where the *OsWOX10* expression was detected after root tip excision in WT (Kawai et al., 2022a). *OsWOX10* was upregulated by IAA treatment (Figure 7E) and revealed as a potential target of the ARF transcriptional activator in the auxin signaling pathway (Figure 7G). Therefore, auxin signaling upregulation in basal part of LRP induces *OsWOX10* expression, increasing LR diameter. Further analysis of spatial-temporal regulation of auxin distribution in LRP under different environmental conditions is required to reveal the detailed mechanisms of LR diameter regulation.

## Conclusion

**FIGURE 8:**
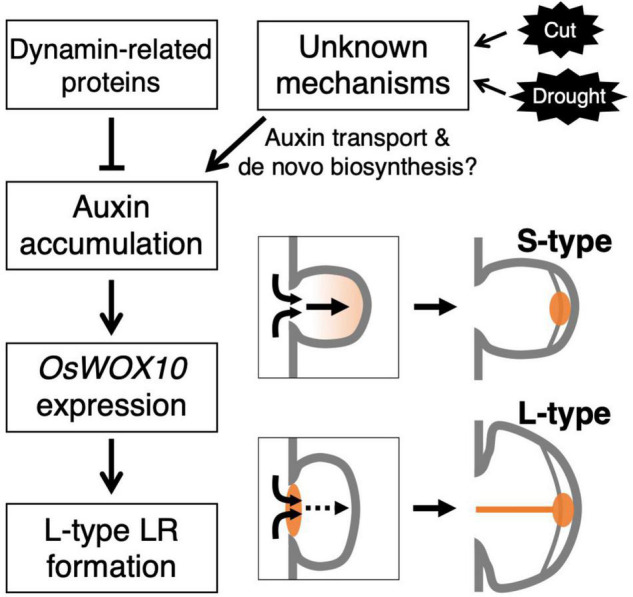
Schematic model showing the regulation of lateral root primordium (LRP) size by auxin distribution. The mutations in genes encoding dynamin proteins resulted in the promoted L-type LR formation with auxin accumulation at basal part of LRP. The accumulated auxin induces a positive regulator of LRP size, *OsWOX10*, promoting L-type LR formation. In the wild-type, unknown mechanisms might induce the auxin accumulation in LRP in response to the root tip excision (Cut) or drought stress.

This study revealed the involvement of auxin distribution in LR diameter control (Figure 8). LR diameter increased in *drp* mutants with the upregulated auxin signaling in LRP including the basal part at an early stage. The *drp* mutants had a decreased endocytic activity with reduced PIN protein localization in endosomal compartments. Inhibiting polar auxin transport by NPA and BFA enhanced the effect of exogenous auxin application on LR diameter increase. The co-treatment of IAA and NPA resulted in auxin accumulation in the basal part of LRP. Therefore, it is conceivable that auxin accumulation at the basal part of LRP increases LR diameter. The increased *OsWOX10* expression was detected by exogenous IAA application and in *drp* mutants. Furthermore, inactivation of auxin signaling by m*OsIAA3-GR* suppressed the L-type LR formation after root tip excision. These data indicate that auxin accumulation in LRP, especially at the basal part, increases LR diameter through *OsWOX10* upregulation.

## Data Availability Statement

The original contributions presented in the study are included in the article/Supplementary Material, further inquiries can be directed to the corresponding author/s.

## Author Contributions

TKa and YI conceived, designed the experiments, and wrote the manuscript. TKa, RA, IS, TKo, MS, and HT performed the experiments. TKa, RA, and YI analyzed the data. All authors contributed to the article and approved the submitted version.

## Conflict of Interest

The authors declare that the research was conducted in the absence of any commercial or financial relationships that could be construed as a potential conflict of interest.

## Publisher’s Note

All claims expressed in this article are solely those of the authors and do not necessarily represent those of their affiliated organizations, or those of the publisher, the editors and the reviewers. Any product that may be evaluated in this article, or claim that may be made by its manufacturer, is not guaranteed or endorsed by the publisher.

## Footnotes

1
https://www.agr.nagoya-u.ac.jp/~shinkan/LIA32

## Abbreviations

AuxRE: Auxin response element
BFA: Brefeldin A
CME: Clathrin-mediated endocytosis
CR: Crown root
DEX: Dexamethasone
DRP: Dynamin-related protein
EMSA: Electrophoresis mobility shift assay
GR: Glucocorticoid receptor
IAA: Indole-3-acetic acid
LR: Lateral root
LRP: Lateral root primordial
MNU: *N*-methyl-*N*-nitrosourea
NPA: *N*-1-naphthylphthalamic acid
SD: Standard deviation
SR: Seminal root
WT: Wild-type.
