# Supplementary material for: Auxin Distribution in Lateral Root Primordium Development Affects the Size and Lateral Root Diameter of Rice
Source: Front Plant Sci. 2022 Apr 13;13:834378. doi: 10.3389/fpls.2022.834378 (PMC9043952; doi:10.3389/fpls.2022.834378)
Supplement: Supplementary file 1 [file Data_Sheet_1.docx]

Supplementary Material

**Figure S1 |** Analysis of root and shoot phenotypes in 10-day-old seedlings of the wild-type (WT) and mutants. **(A)** Density of lateral roots (LRs). **(B, C)** Means of LR length in different diameter classes. D., LR diameter. **(D)** Seminal root length. **(E)** Crown root number. **(F)** Length of second leaf. Values represent mean ± SD (*n* = 8, 5 independent biological replicates in **A, D–F** and **B, C**, respectively). Different letters indicate significant differences among genotypes (*P* < 0.05).

**Figure S2 |** Complementation test of T12-3. **(A, B)** Regenerated plants of T12-3 with the **(A)** empty vector and **(B)** *pOsDRP1C*-*OsDRP1C*-*GFP* construct. **(C–E)** LR phenotypes on crown roots in the **(C)** vector control and **(D, E)** complemented line. Scale bars = 1 cm.

**Figure S3 |** Expression pattern of **(A)** *OsDRP1C* and **(B)** *OsDRP2B* in various tissues and organs throughout entire growth in a rice gene expression profile database (RiceXPro; Sato et al., 2013b).

**Figure S4 |** Expression analysis of *OsDRP1C* and *OsDRP2B* in seminal roots (SRs) and lateral root primordia (LRP) in wild-type. **(A–C)** Expression analysis along SR axis. **(A)** Schematic diagram showing sample regions in SR. Expression levels of **(B)** *OsDRP1C* and **(C)** *OsDRP2B* in different regions. Values represent mean ± SD (*n* = 3 biological replicates). Different letters indicate significant differences among groups (*P* < 0.05). **(D)** Expression of *DRP* family genes in rice in S- and L-type LRP. Value represents mean of ‘Transcripts Per Million’ (TPM) in the RNA-seq analysis (*n* = 4 biological replicates). No significant difference was detected between S- and L-type LRP in each gene (*Padj* ≥ 0.05).

**Figure S5 |** Expression of *PIN* family genes in rice in S- and L-type LRP. Value represents mean of ‘Transcripts Per Million’ (TPM) in the RNA-seq analysis (*n* = 4 biological replicates). No significant difference was detected between S- and L-type LRP in each gene (*Padj* ≥ 0.05). Accession numbers of genes are in **Table S4**.

**Figure S6 |** Analysis of the effect of exogenous auxin and chemical inhibitors on diameter of lateral root (LR) on seminal roots in wild-type (WT) and mutants. (**A–D**) Maximum LR diameter in **(A)** IAA and NPA, **(B)** IAA, **(C)** IAA and BFA, and **(D)** 2,4-D treatment in the WT. Values represent mean ± SD (*n* = 5 independent biological replicates). Different letters indicate significant differences among groups (*P* < 0.05). (**E**) Density of thicker LRs (≥150 μm) in the WT and mutants. D., LR diameter. Values represent mean ± SD (*n* = 5 independent biological replicates) (**p* < 0.05; ***p* < 0.01; n.s., not significant).

|  | | Forward | | Reverse |
| --- | --- | --- | --- | --- |
| **Map-based cloning** | | |  | |
|  | RM15780 | ACCTTCGACGCTATCAGATTTGG | | ATAGCAAAGGAGTCGCAAAGACC |
|  | RM15796 | AAGCCTAACGGCAGCGAAAGC | | GACGTCGTACTCGTGCCTCACC |
|  | RM15804 | CATCCACTTCCTTGATCTTGTAGC | | TGCTTAGCCGCTACACTACTTCC |
|  | RM15805 | CACTAACGGTGTCACTGCTTGC | | ACCTCTTCCATCCCTCCTTTCG |
|  | RM15812 | CCCATCGCCACTATATACATACCC | | AAACGGAGAAAGAAAGAGGAGAGG |
|  | RM15414 | TCCATCATATGCTCTGCTCTCTGC | | CCTCCCTTCCTCCAGATCACC |
|  | RM3199 | TAAAAACCTCACCTCGCTGG | | TTCGTTCACTCAGTGGCTTG |
|  | RM5472 | CACTCAAGACCAGACCTGTACG | | CGGCACGTCATTGTAGTGAC |
|  | RM240 | CCTTAATGGGTAGTGTGCAC | | TGTAACCATTCCTTCCATCC |
|  | RM13941 | CACTCAAGACCAGACCTGTACG | | CGGCACGTCATTGTAGTGAC |
|  | RM13949 | TCTCCCTCTTCTACTAATGCTACC | | GGGAAAGGATTGAGAAGATACG |
| **Gene expression** | | |  | |
|  | *OsDRP1C* | GCGTTTCACTGACTTTGCTG | | TGTCAGGTTTACAACATGTGGAG |
|  | *OsDRP2B* | GATCCAGATGGAACCAGAAC | | GCAACCCACTCAATTTCAGC |
|  | *OsWOX10* | ACCACCATTACAGCAGCTACG | | GACGCCATTGATGAACACTTGG |
|  | *OsUBQ5* | AACCAGCTGAGGCCCAAGA | | ACGATTGATTTAACCAGTCCATGA |
| **Vector construction** | | |  | |
| **(Complementation test)** | | | | |
|  | *OsDRP1C* genome | AAAAGCAGGCTCCGCCGGCTACCTTAATTTGCTTT | | AGGCTCCCCATCGTCGCCAT |
|  | *OsDRP1C* CDS | ATGGCGACGATGGGGAGCCT | | AGAAAGCTGGGTCGGCTTTCCACGCGACTGAGTCG |
| **(Transient expression)** | | | | |
|  | *OsPIN1b* CDS | ATTTACAATTTCTAGATGATTACGGCGGCGGACTT | | AGAAAGCTGACAGCCCAAGCAAGATGTAGT |
|  | *GFP* | GCTTGGGCTGTCAGCTTTCTTGTACAAAGT | | GATCGGGGAAATTCGTTACTTGTACAGCTCGTCCA |
|  | *35S*-*OsPIN1b*-*GFP* | GCAGGCTCCGCGGCCAAGCTTGCGTATTGGCTAGA | | AGCTGGGTCGGCGCGCATGATTACGAATTGGTTCC |
| **(EMSA)** | | | | |
|  | *OsARF19* DB | TATCGGATCCGAATTAATGATGAAGCAAGCTCAACA | | GACGGAGCTCGAATTTTATGAGGATGAAATGTTCGTCG |

**Table S1 |** List of primers.

| (A) *OsDRP1C* |  |  |  |
| --- | --- | --- | --- |
| Locus ID | Description | Mutual Rank | Weighted PCC |
| Os07g0200000 | Conserved hypothetical protein. | 2 | 0.829098 |
| Os02g0805000 | Adaptin, N-terminal domain containing protein. | 3.464 | 0.787272 |
| Os02g0209000 | WD40-like domain containing protein. | 4 | 0.782668 |
| Os01g0744400 | Conserved hypothetical protein. | 4 | 0.751904 |
| Os10g0512700 | Similar to SH3 domain-containing protein 3. | 4.243 | 0.772771 |
| Os02g0738900 | **Similar to Dynamin 2B (EC 3.6.5.5) (Dynamin-related protein 2B) (Dynamin-like protein 3).** | 5.196 | 0.750071 |
| Os07g0604800 | Similar to Alpha-1,4-glucan-protein synthase [UDP-forming] 1 (EC 2.4.1.112) (UDP- glucose:protein transglucosylase 1) (UPTG 1). | 5.477 | 0.749753 |
| Os02g0810500 | RabGAP/TBC domain containing protein. | 7.071 | 0.717768 |
| Os11g0183800 | Non-protein coding transcript, unclassifiable transcript. | 7.211 | 0.737204 |
| Os08g0155100 | Major sperm protein domain containing protein. | 7.55 | 0.724368 |

**Table S2 |** Co-expression genes of **(A)** *OsDRP1C* and **(B)** *OsDRP2B* in a rice functional related gene expression network database (RiceFREND; Sato et al., 2013a). Top 10 co-expressed genes are shown for each gene.

**Table S2 |** Continued.

| (B) *OsDRP2B* |  |  |  |
| --- | --- | --- | --- |
| Locus ID | Description | Mutual Rank | Weighted PCC |
| Os02g0552000 | Similar to Transmembrane protein TM9SF3 (Fragment). | 3.162 | 0.759394 |
| Os12g0443600 | Similar to UDP-glucose 6-dehydrogenase (EC 1.1.1.22) (UDP-Glc dehydrogenase) (UDP-GlcDH) (UDPGDH). | 4.472 | 0.698709 |
| Os03g0713100 | **Similar to Dynamin-related protein 1C (Dynamin-like protein C) (Dynamin-like protein 5) (Dynamin-like protein DLP1).** | 5.196 | 0.750071 |
| Os02g0209000 | WD40-like domain containing protein. | 6.928 | 0.734018 |
| Os03g0737800 | Conserved hypothetical protein. | 6.928 | 0.682834 |
| Os07g0604800 | Similar to Alpha-1,4-glucan-protein synthase [UDP-forming] 1 (EC 2.4.1.112) (UDP- glucose:protein transglucosylase 1) (UPTG 1). | 8.944 | 0.701942 |
| Os06g0669600 | Hypothetical protein. | 9.22 | 0.721974 |
| Os01g0744400 | Conserved hypothetical protein. | 9.899 | 0.687809 |
| Os02g0644400 | Hypothetical protein. | 10.247 | 0.685081 |
| Os02g0805000 | Adaptin, N-terminal domain containing protein. | 10.392 | 0.708557 |

| Name | Gene ID | Accession number (UniProt) |
| --- | --- | --- |
| *AtDRP1A* | AT5G42080 | P42697 |
| *AtDRP1B* | AT3G61760 | Q84XF3 |
| *AtDRP1C* | AT1G14830 | Q8LF21 |
| *AtDRP1D* | AT2G44590 | Q8S3C9 |
| *AtDRP1E* | AT3G60190 | Q9FNX5 |
| *AtDRP2A* | AT1G10290 | Q9SE83 |
| *AtDRP2B* | AT1G59610 | Q9LQ55 |
| *AtDRP3A* | AT4G33650 | Q8S944 |
| *AtDRP3B* | AT2G14120 | Q8LFT2 |
| *AtDRP4A* | AT1G60530 | Q9ZP56 |
| *AtDRP4C* | AT1G60500 | Q9ZP55 |
| *AtDRP5A* | AT1G53140 | F4HPR5 |
| *AtDRP5B* | AT3G19720 | Q84N64 |
| *OsDRP1A* | Os05g0556100 | Q0DG31 |
| *OsDRP1B* | Os01g0681100 | A0A0P0V6L3 |
| *OsDRP1C* | Os03g0713100 | Q8W315 |
| *OsDRP1D* | Os10g0567800 | Q7XBZ9 |
| *OsDRP1E* | Os09g0572900 | Q650Z3 |
| *OsDRP2A* | Os06g0247800 | Q654U5 |
| *OsDRP2B* | Os02g0738900 | Q0DXR0 |
| *OsDRP2C* | Os08g0425100 | Q0J5L5 |
| *OsDRP3A* | Os01g0920400 | Q5JJJ5 |
| *OsDRP3B* | Os04g0381000 | A0A0P0W9A2 |
| *OsDRP4A* | Os03g0260000 | Q10NS9 |
| *OsDRP4B* | Os04g0129900 | A0A0P0W699 |
| *OsDRP5A* | Os01g0748000 | Q0JJC7 |
| *OsDRP5B* | Os12g0178700 | Q0IPN9 |

**Table S3 |** List of *DRP* family genes in *Arabidopsis* and rice.

| Name | Gene ID |
| --- | --- |
| *OsPIN1a* | Os06g0232300 |
| *OsPIN1b* | Os02g0743400 |
| *OsPIN1c* | Os11g0137000 |
| *OsPIN1d* | Os12g0133800 |
| *OsPIN2* | Os06g0660200 |
| *OsPIN5a* | Os01g0919800 |
| *OsPIN5b* | Os08g0529000 |
| *OsPIN5c* | Os09g0505400 |
| *OsPIN8* | Os01g0715600 |
| *OsPIN9* | Os01g0802700 |
| *OsPIN10a* | Os01g0643300 |
| *OsPIN10b* | Os05g0576900 |

**Table S4 |** List of *PIN* family genes in rice.
